# “We are everything to everyone”: a systematic review of factors influencing the accountability relationships of Aboriginal and Torres Strait Islander health workers (AHWs) in the Australian health system

**DOI:** 10.1186/s12939-018-0779-z

**Published:** 2018-05-30

**Authors:** Stephanie M. Topp, Alexandra Edelman, Sean Taylor

**Affiliations:** 1 College of Public Health, Medical and Veterinary Sciences, James Cook University, James Cook Drive, Townsville, QLD 4810 Australia; 2 Nossal Institute for Global Health, University of Melbourne, Melbourne, VIC 3010 Australia; 3 Torres and Cape Hospital and Health Service, Community Wellness Centre, Thursday Island Hospital Campus, Thursday Island, QLD 4875 Australia

**Keywords:** Community health workers, Aboriginal and Torres Strait Islander, Health system, Accountability, Governance, Power relations, Universal health coverage

## Abstract

**Background:**

Health policy in Australia positions Aboriginal and Torres Strait Islander Health Workers (AHWs) as central to improving Aboriginal and Torres Strait Islander peoples’ health, with high expectations of their contribution to closing the gap between Indigenous and non-Indigenous health outcomes. Understanding how AHWs’ governance and accountability relationships influence their ability to address such health inequities has policy, programme and ethical significance. We sought to map the evidence of AHWs’ experiences of accountability in the Australian health system.

**Methods:**

We followed an adapted qualitative systematic review process to map evidence on accountability relations in the published literature. We sought empirical studies or first-person accounts describing AHWs’ experiences of working in government or Aboriginal community-controlled services anywhere in Australia. Findings were organised according to van Belle and Mayhew’s four dimensions of accountability – social, political, provider and organisational.

**Results:**

Of 27 included studies, none had a primary focus on AHW governance or AHWs’ accountability relationships. Nonetheless, selected articles provided some insight into AHWs’ experiences of accountability across van Belle and Mayhew’s four dimensions. In the social dimension, AHWs’ sense of connection and belonging to community was reflected in the importance placed on AHWs’ cultural brokerage and advocacy functions. But social and cultural obligations overlapped and sometimes clashed with organisational and provider-related accountabilities. AHWs described having to straddle cultural obligations (e.g. related to gender, age and kinship) alongside the expectations of non-Indigenous colleagues and supervisors which were underpinned by ‘Western’ models of clinical governance and management. Lack of role-clarity stemming from weakly constituted (state-based) career structures was linked to a system-wide misunderstanding of AHWs’ roles and responsibilities – particularly the cultural components – acting as a barrier to AHWs working to their full capacity for the benefit of patients, broader society and their own professional satisfaction.

**Conclusions:**

In literature spanning different geographies, service domains and several decades, this review found evidence of complexity in AHWs’ accountability relationships that both affects individual and team performance. However, theoretically informed and systematic investigation of accountability relationships and related issues, including the power dynamics that underpin AHW governance and performance in often diverse settings, remains limited and more work in this area is required.

## Introduction

Globally, community health workers (CHWs) have a long history in health systems. In the years immediately after the landmark Alma Ata Conference of 1978, CHWs were lauded as an important pillar of primary health care. Indeed, there is now well-established evidence on the role of CHWs and community-based health action in supporting improved health outcomes, particularly for marginalised or vulnerable populations [1, 2]. Yet the governance and leadership challenges surrounding the integration of CHWs into national health systems are complex and not all efforts have been successful. As early as 1981, community health advocates were asking whether CHWs were truly positioned to be liberators capable of enabling citizen participation in health through individual and communal empowerment, or, whether they were simply lackeys for an over-burdened health system [3]? Nearly forty years later, this question is still relevant, as community health workers – including Australia’s Aboriginal and Torres Strait Islander Health Workers (AHWs) – are being placed in pivotal roles as part of national programs that seek to accomplish universal health coverage, or in Australia’s case ‘close the gap’ between Indigenous and non-Indigenous health outcomes [4, 5].

The role of AHWs evolved in northern Australia during the 1950s. Initially employed as leprosarium workers and later as medical assistants in the Northern Territory [6–8], AHWs became important members of primary health services Australia-wide through the 1970s and 80s; this paralleled the development of Aboriginal community controlled health services who championed their role [9]. Initial conceptions of the AHW role were similar to that of community or primary healthcare workers in the international context with a focus on primary health care tasks such as health education, basic health care, and community health action. But as the AHW role developed in the Northern Territory, a notable feature of AHW practice became the emphasis placed on ‘cultural brokerage’ and the focus on provision of culturally safe and comprehensive primary health care services to Aboriginal and Torres Strait Islander people [7, 8]. These features are particularly important in both the historical and contemporary contexts in which AHWs work, in which Aboriginal and Torres Strait Islander populations have experienced, and continue to experience, a high burden of disease and poor access to mainstream government health services [4]. Indeed, the cultural component of AHWs’ work, which includes helping to foster community trust and wellbeing, is now embodied in policy definitions of ‘AHWs’ [4, 8, 10] and the profession is explicitly linked to self-identification and community recognition as an Aboriginal and/or Torres Strait Islander person. The culturally constituted component of the AHW role clearly distinguishes it from other Australian health professions, but is not necessarily unusual within the broad spectrum of CHW roles internationally [11].

### Training and registration

AHWs currently require a minimum qualification of a Certificate III in Aboriginal and/or Torres Strait Islander Primary Health Care. Aboriginal and Torres Strait Islander Health *Practitioners* (AHPs) are the registered members of the AHW workforce. National Registration for AHPs was introduced in 2012 under the National Registration and Accreditation Scheme (NRAS) administered by the Aboriginal and Torres Strait Islander Health Practice Board of Australia. To achieve registration and practitioner status, an individual must hold a minimum Certificate IV in Aboriginal and/or Torres Strait Islander Primary Health care with the option to specialise in either community care or clinical care. Various technical and further education (TAFE) institutions or specialist colleges (e.g Batchelor Institute) around Australia provide Certificate III and IV qualifications. Multiple options for further specialisation include mental health, family health, sexual health, health education, hospital liaison, drug, and alcohol services [4, 8]. AHWs (encompassing AHPs) may be employed by state or territory health departments to work in government hospitals or primary care services; by Aboriginal community controlled health services; or by other entities delivering health and allied services. Remuneration is dependent on the employing organisation, but AHWs and AHPs are typically among the lowest paid in both government and non-government organisations [12, 13].

Robust and up-to-date data on the AHW workforce is lacking. Among all Australian states and territories, only the Northern Territory maintains a comprehensive database of AHWs who, under Northern Territory law, must all achieve registration as a practitioner in order to be employed. Based on census data, however, there were 1256 AHWs Australia-wide in 2011, double the number of AHWs recorded in 1996 [4]. Of these, 72% (*n* = 908) were female, and just over half (56%) were trained to a certificate IV level or higher, and thus eligible for registration. Eighty nine AHWs (7%) had a bachelor’s degree or higher in 2011 [4].

### Challenges, Tensions & Gaps in knowledge

Health policy in Australia continues to position the AHW workforce as central to improving Aboriginal and Torres Strait Islander peoples’ health (see for instance Table 1), with high expectations of the role’s contribution to the Close the Gap agenda and achievement of universal health coverage [5, 14]. Yet, as is being experienced in CHW programmes in other countries [1, 15–17] serious challenges exist in relation to the implementation and governance of the AHW profession. Lack of state or national scopes of practice for example, have resulted in pressure on individual AHWs to fulfil ambitious localised terms of reference without sufficient support, supervision or resourcing [18, 19]. Challenges relating to the ways in which AHWs balance (sometimes competing) obligations to their communities and their clinical service managers also exist [4, 12]. Such issues appear to be contributing to difficulties in both recruitment and retention, with substantial numbers of unfilled AHW posts nationally pointing to ongoing and potentially intensifying issues of job satisfaction [8] that bring the very sustainability of the role into question.

**Table 1:** Summarised policy timeline in the development of the AHW role

Year	Key developments
1950s – 1990s	• The first AHW roles commenced with the employment of Aboriginal women as leprosarium workers and hospital assistants in Northern Territory in the 1950s and 60s. • Development of the role in the 1970s and 80s followed national recognition of the need for an accessible and culturally safe workforce to address government or ‘mainstream’ health service gaps for Aboriginal and Torres Strait Islander peoples, and adopted a primary health care focus. • Australia’s first Aboriginal community controlled health service was established in Redfern in 1971, with services subsequently established Australia-wide. The developing community-controlled sector championed the role of AHWs as key members of multidisciplinary primary healthcare teams. • AHWs were recognised as a professional group in the Northern Territory through the *Northern Territory Health Practitioners and Allied Health Professionals Registration Act 1985*, which addressed restrictions on entry, registration, title, practice and disciplinary provisions. • National competency standards were developed for AHWs in 1996, although these were not universally adopted.
2000s – current	• A 2000 report, *Training Re-Visions: A National Review of Aboriginal and Torres Strait Islander Health Worker Training* by the Commonwealth Office for Aboriginal and Torres Strait Islander Health addressed AHW training priorities following a national review. • The National Aboriginal and Torres Strait Islander Health Worker Association (NATSIHWA) was established in 2009 as the peak national body for AHWs and Practitioners in Australia, following Australian Government commitments to strengthen the AHW workforce as part of ‘Closing the Gap’. • The *National Aboriginal and Torres Strait Islander Health Workforce Strategic Framework (2011–2015)* prepared for the Australian Health Ministers’ Advisory Council, emphasised the role of AHWs in achieving equitable health outcomes for Aboriginal and Torres Strait Islander peoples. • Health Workforce Australia’s *National Health Workforce Innovation and Reform Strategic Framework for Action (2011–2015)* emphasised the need to increase the number of AHWs working in the health sector to improve Aboriginal and Torres Strait Islander health. • Health Workforce Australia’s *Aboriginal and Torres Strait Islander Health Worker Project Final Report (‘Growing Our Future’)* released in 2011 articulated policies and strategies to strengthen and sustain the AHW workforce in Australia. • From 1 July 2012, AHWs who have gained Certificate IV in Aboriginal and/or Torres Strait Islander Primary Health Care Practice can register as an Aboriginal and Torres Strait Islander Health Practitioner under the National Registration and Accreditation Scheme. AHWs are not required to register unless deemed necessary for employment purposes. Health Practitioners previously regulated by the NT Boards are now regulated under the national Scheme. • The *National Aboriginal and Torres Strait Islander Health Workforce Strategic Framework (2016–2023)*, developed within the context of the *National Aboriginal and Torres Strait Islander Health Plan 2013–2023*, guides Aboriginal and Torres Strait Islander health workforce policy to build a strong and supported health workforce capable of providing culturally-safe and responsive health care.

Access to healthcare for socially marginalised or vulnerable groups, including Aboriginal and Torres Strait Islander peoples, is mediated by the governance of services [20, 21]. Drawing on public administration and development theory, health systems researchers increasingly acknowledge that governance of health services should be understood not simply as the formal rules and infrastructure enabling health service delivery, but as the complex network of relationships and informal (practical) norms that arise from constant interaction among health workers, and between health workers and their clients [21–24]. Cleary et al. [23] among others have noted that at the sub-national level, governance approaches must enable and sustain service responsiveness, including by promoting system learning and accountability. Accountability for health, as a feature of a health system, is attained when governments respect, protect, and fulfill the right to health, and when health sector employees are treated respectfully. Yet despite their importance, exploration of these concepts and experiences of health service governance and accountability (NB: not clinical governance) are still emerging fields and a largely neglected area of research in relation to Aboriginal and Torres Strait Islander health care in Australia.

### Conceptual framework: Governance and accountability

Following recent public administration and health systems scholars [22, 23, 25, 26], we understand accountability as one lens that may help improve our understanding of aspects of AHW governance and performance. Although used variably in different disciplines, contemporary scholarship generally frames accountability as dispersed, relational and multi-directional [26]. In the context of health systems and health services, this emphasis on the dispersed and multi-directional nature of accountability is critical given the multiple individual and institutional stakeholders networked across multiple levels of service administration and delivery. ‘Vertical’ or ‘bureaucratic’ accountability, referring to service providers’ accountability to their government employer, thus co-exists and interacts with horizontal accountability (intra- and inter-organisational relationships at the same level) and ‘social’, ‘political’ or ‘downwards’ accountability (flowing from funders, planners and providers towards service users or citizens). Haloran [27] and Fox et al. [28] describe these relationships as existing within an ‘accountability eco-system’, a term designed to draw attention to their co-existence and interactive and sometimes inter-dependent nature.

Van Belle and Mayhew [26, 29] posit that there are four dimensions of accountability in local health systems, including health services, namely – provider, organisational, political and social accountability. The *provider* dimension focuses on health workers’ relationship with their direct clients. The *organisational* dimension focuses on how responsive an organisational unit – e.g. a health facility or district health service – is to its stakeholders more broadly, encompassing internal relationships among staff, and upwards and downwards relationships to catchment communities and funders. The *political* dimension of accountability examines regulatory and higher-order management relationships. Finally, the *social* dimension is described as focusing on relationships that enhance equity and social justice. The choice of this framework was deliberate and based on its non-prescriptive nature, which made it appropriate for exploratory work. The framework acknowledges overlap and interaction between the four dimensions, but provides a useful starting point for analysis of the different ‘flows’ of accountability experienced by AHWs. Using a systematic qualitative literature review, this paper thus seeks to explore the state of evidence about the way accountability relations in these different dimensions are experienced by AHWs in Australia.

## Methods

This review was designed to map the available evidence base. The process followed standard qualitative systematic review steps [30–33] but adapted quality criteria to be more inclusive. Since the data accessed were publicly available and previously published, formal ethical considerations and confidentiality procedures were not required.

### Search strategy and selection criteria

We sought examples of empirical studies or first-person accounts describing AHWs’ experiences of working in government or Aboriginal community-controlled health services anywhere in Australia. No date restrictions were applied. No language restrictions were applied; however we anticipated papers reporting on AHWs would be exclusively reported in English. Due to the difficulty in ensuring a systematic approach, grey literature were excluded from the formal search. Included studies reported either directly or indirectly on AHWs and equivalent roles (e.g. Strong Women Workers) delivering care to Aboriginal and/or Torres Strait Islander Australians in any health care setting in Australia. We excluded quantitative studies in which the AHW ‘voice’ was absent. AHWs’ experiences of accountability had to be described through AHWs’ own reflections on the nature and challenges of their roles. Studies that failed to obtain or report appropriate ethical approvals were excluded. We also excluded studies reporting on AHW-led programs if they included no meaningful data about AHW experiences within those programs. Although not empirical studies, several commentaries authored or co-authored by AHWs reflecting directly on personal experiences in the workplace were included as highly authoritative accounts of relevance to the review topic.

We searched MEDLINE, CINAHL, Scopus and Informit (health suite and social sciences suite) for peer-reviewed publications and dissertations and theses using the following terms: (“health worker” OR “health practitioner” OR “health professional”) AND (Austral* OR “New South Wales” OR “Northern Territory” OR Queensland OR Victoria OR Tasmania) AND (Indigenous OR Aborigin* OR “Torres Strait Islander” OR “oceanic ancestry group” OR “oceanic ancestry groups” OR australoid* OR “Australian race” OR “cultural competence” or “cultural competencies”). Subject terms were also used in the searches in Medline and CINAHL. Search terms were refined in consultation with a university librarian.

**Fig. 1:**
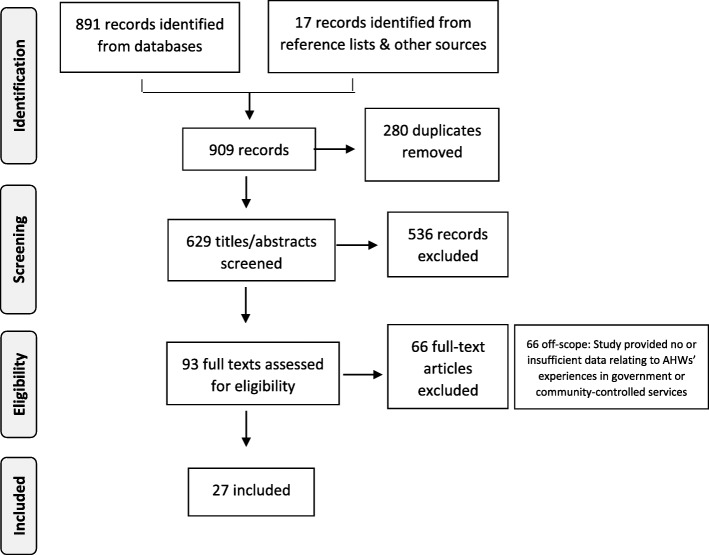
Literature search and study selection

Following a number of pilot searches, we removed the search terms ‘accountability’, ‘transparency’, ‘answerability’ and ‘social responsibility’, as they introduced a large number of irrelevant (non AHW-related) records while yielding no new records of interest for the review. The terms used in the final searches enabled identification of a full range of articles incorporating AHW experiences and practice for consideration against other criteria. Duplicates were removed and titles and abstracts were screened by the first and second author against inclusion criteria. Two authors independently reviewed full texts of all included articles with disagreement resolved by consensus (Fig. 1). Hand searches were subsequently conducted of the reference lists of selected articles as well as of the Aboriginal and Islander Health Worker journal, applying the same selection criteria.

### Analysis

We used thematic analysis to identify and categorise the data from selected articles using van Belle and Mayhew’s typology of accountability dimensions as an overarching guide. Close and repeated reading of the papers allowed identification of specific experiences directly or indirectly related to AHWs accountability relationships. For example, we looked for findings reporting or discussing AHW responsibilities, experiences and perceptions of issues such as training, supervision, respect, recognition, appreciation, scope of work, team work, power relations, autonomy and decision making authority, feedback mechanisms and resourcing. This was possible since included articles were required to describe AHWs’ self-reported experiences and perceptions. Where available, we also extracted data on the perceptions of professional health workers and community members. Such experiences formed the basic codes that enabled data extraction. First level codes were grouped into grounded themes, and subsequently organised according to their relevance against the four dimensions of accountability synthesised by van Belle and Mayhew [26]. We followed an iterative process of reflection on the appropriateness of the mid-level themes, and their categorisation under the four dimensions, resulting in several rounds of adaptation and re-grouping. Interpretation of the AHW experience was conducted using an overarching lens of intersectionality, which has regard for the intersecting identities and experiences of the Aboriginal and Torres Strait Islander peoples’ health workforce. This lens enabled us to consider how potentially interlocking systems of power within Aboriginal and Torres Strait Islander communities, in clinic settings, and across the broader health service domain were impacting on AHWs’ ability to fulfil an ambitious policy remit.

### Limitations

Articles included in this review were published or readily available studies, commentaries books and reports. Several older government reports (c.1980) found in the reference lists of included articles could not be accessed, but these are unlikely to have reported AHW perspectives directly, as was required for inclusion in this study. As the findings of this review reflect the data found in the included publications, the importance of some issues may be unintentionally under- or over-emphasised. We have attempted to be as transparent as possible in reporting of the origins and focus of included works as well as in highlighting a range of gaps in knowledge that this review has identified. As the purpose of this review was to map the evidence base relating to AHW accountability, selected articles were not assessed for the quality (as in standard systematic reviews) beyond an assessment of their relevance and depth of analysis with regards to issues of AHW governance and accountability. Finally, a note on nomenclature: we predominantly use ‘Aboriginal and Torres Strait Islander peoples’, but ‘Indigenous Australian’ is used where the review discusses findings of included papers that use this alternative terminology; both are widely used nomenclature to describe Australia’s First Nation’s peoples.

## Findings

### Characteristics of selected articles

Our findings are based on 27 studies published between 1995 and 2017 (Table 2). Twenty four studies were reported in peer-reviewed journal articles, the majority of which (*n* = 21) used qualitative research designs employing semi-structured interviews with AHWs and other staff and/or patients. The remaining three peer-reviewed papers were commentary or opinion pieces written by AHWs. One thesis, one monograph, and a book were also included. For ease of reference, we use the generic term ‘papers’ when referring to all the above sources.

**Table 2:** Summary and Key Data Extracted from Review Articles

Author (date)	Title	Journal/publication	Study type & methods	Focus location & organisational setting	Key themes relating to AHWs’ accountability relationships in the four domains
Tregenza, J. & Abbott, K. 1995 [41]	Rhetoric and reality: Perceptions of the roles of Aboriginal Health Workers in Central Australia	Monograph	Empirical: intersectional lens using mixed methods; semi-structured interviews;. 294 AHWs from 26 communities.	26 Remote and Regional communities in Central Australia (Northern Territory)	Social • AHWs ‘agents’ to improve health status and agents of social change in the community • Being Aboriginal and part of the community gives strength in role Provider • AHWs seen as main providers of healthcare in the community Political/Organisational • Clear power differentials with non-Aboriginal colleagues, with AHWs are ‘bottom of the rung’ • Perception that further education could enhance power and status • Non-Indigenous co-workers’ & supervisors’ perceptions of role frequently divergent with their own and a source of tension
McMasters, A., 1996 [39]	Research from an Aboriginal health worker’s point of view	Australian & New Zealand Journal of Public Health	Non-empirical: experiential/opinion	Central Australia	Political • High expectations of AHW by mainstream health system Political/Organisational • Demanding responsibilities of the role, compounded for AHWs from remote communities, at odds with levels of knowledge and power • Tensions between Western and Indigenous models of health promotion
Hecker, R. (1997) [52]	Participatory action research as a strategy for empowering aboriginal health workers	Australia New Zealand Journal of Public Health	Empirical: empowerment lens using participatory action research	ACCHO in Pitjantjatjara Lands, remote South Australia	Political • Access to practical training ad hoc Organisational • Perception that opinions not valued and no representation on key health service committees
Jackson, D. et al., 1999 [40]	Towards (re)conciliation: (re)constructing relationships between Indigenous health workers and nurses	Journal of Advanced Nursing	Empirical: feminist lens, qualitative descriptive; in-depth interviews	Undisclosed Australian health service settings	Political • Role confusion involving inconsistent definitions of role and scope Organisational/Political • Power dynamics interfere with capacity for cross-professional collaboration • Cultural advocacy role key but un- or under-recognised by other professions Provider • Complex lines of responsibility to local community, family and bio-medically oriented health services
Dollard, J.S. et al., 2001 [51]	Aboriginal Health Worker Status in South Australia	Aboriginal and Islander Health Worker Journal	Empirical: qualitative descriptive; questionnaire (*n* = 74), in depth interviews (10), and 4 focus group discussions (*n* = 35)	Various health service settings in South Australia	Political/Organisational • Lack of satisfaction with status compared to other staff linked to lack of professional and role recognition; inequality compared to other professionals (e.g. pay and qualifications required for appointments) • Limited capacity to voice concerns at high levels due to vertical power structures Organisational • Perception of being ‘jack of all trades but master of none’
Williams, C. 2003 [46]	Aboriginal health workers, emotional labour, obligatory community labour and occupational health and safety	Journal of Occupational Health and Safety Australia and New Zealand	Empirical: Sociological lens placing AHWs within institutional context; qualitative descriptive; interviews of 29 AHWs	Variety of health service settings, South Australia	Social • Experience of strong sense of obligation to care for family and kin of patient as well as patient • Community advocacy and cultural brokerage role crucial to AHWs Organisational • AHWs seen as point of contact for all Aboriginal patients regardless of health problem - causing tensions due to conflicting understandings of role Political/Organisational • Experiences of racism from non-Aboriginal co-workers
Genat, B. (2006) [7]	Aboriginal Health workers: Primary Health Care at the Margins.	Book	Empirical: Ethnographic based on 6 AHWs experiences & interviews with colleagues and community clients in late 1990s	Urban Aboriginal Community Controlled Service in Western Australia	Social • AHWs experience strong sense of obligation to Aboriginal clients but tensions around perception that they should be available 24/7 Political/Organisational • AHWs lack voice and status within organisation and sector, that undermines professional capacity Provider • Professional identity of AHWs is tenuous, undermined by weak understanding of the role by colleagues and even clients
Mitchell, M. et al., 2006 [38]	The Aboriginal health worker	Medical Journal of Australia	Non-empirical: experiential/opinion	Aboriginal Community Controlled Organisation, Townsville, Queensland	Political • Challenges in cross-jurisdictional variation in definitions of role, competencies and skills recognition *Organisational* • Different scope of work for AHWs in ACCHO setting compared with mainstream health service setting - reflecting a ‘social model of health’ versus a ‘disease model of health’ Social • Experience of being ‘everything to everyone’, incorporating community demand and expectation for after work hours Organisational • Perceptions of co-workers limited understanding of AHW role and impact on teamwork Provider/Organisational • Reflections on intersection of cultural and social norms relating to age and clan/family groups with workplace dynamics and their impact on patient relationships
Harris, A. et al. (2007) [42]	The Aboriginal Mental Health Worker Program: The challenge of supporting Aboriginal involvement in mental health care in the remote community context	Australian e-journal for the Advancement of Mental Health	Empirical: qualitative descriptive; audits of client records, participant observation, and semi-structured interviews	Remote community health centres, Northern Territory	Political • Lack of consensus on AHWs role in clinical settings Organisational • Role confusion and varied expectations of AHWs • GPs resisting responsibility for proactive mentoring role • Assumption that AHWs are universally culturally skilled, not requiring formal support or development Provider • Role ambiguity and unclear cultural legitimacy source of individual strain and ‘burnout’
Hooper, K. et al., 2007 [43]	Health professional partnerships and their impact on Aboriginal health: an occupational therapist’s and Aboriginal health worker’s perspective	Australian Journal of Rural Health	Empirical: qualitative descriptive; in-depth interviews	Aboriginal and mainstream health and human service organisations in rural and remote North Queensland	Political • Lack of role clarity a barrier to communication and service planning Provider • Cultural advocacy and brokerage role central to efficacy of AHWs • Not having an AHW trained in OT undermines continuity of care
Abbott, P. et al., 2008 [6]	Expanding roles of Aboriginal health workers in the primary care setting: seeking recognition	Contemporary Nurse	Non-empirical: commentary with mini-cases	Australia-wide, with mini-cases focused on an AMS in Western Sydney	Social • Central nature of Cultural brokerage to role Provider • Rarely ‘off duty’ Political • Lack of recognition and limited career opportunities Organisational • Experience more autonomy within community controlled health services
Stamp, G.E. et al., 2008 [50]	Aboriginal maternal and infant care workers: partners in caring for Aboriginal mothers and babies	Rural and Remote Health	Empirical: qualitative descriptive; semi-structured interviews with 5 AMIC workers and 4 midwives	Regional South Australia	Social • Community advocacy perceived to be crucial part of AHW role Organisational • Two-way partnership model with midwives emphasising mutual equivalence and valuing cultural knowledge of AHWs builds community trust in service Political/Organisational • Overcoming initial staff resistance to new AHW roles and agency
Taylor, K.et al., 2009 [47]	Exploring the impact of an Aboriginal Health Worker on hospitalised Aboriginal experiences: lessons from cardiology	Australian Health Review	Empirical: qualitative descriptive; open-ended interviews with 4 cardiology nurses, 3 nurses, 2 doctors, 2 social workers, 2 AHWs, 12 recent Aboriginal clients	Cardiology department in a tertiary hospital, Western Australia	Social • Community advocacy and brokerage work crucial to helping Aboriginal patients Provider • AHWs seen as point of contact for all sociocultural needs of Indigenous patients Organisational • Other staff tend to understand AHW role purely in terms of social and education functions despite clinical training, capacity and interest
Lloyd, J. et al., 2009 [45]	The influence of professional values on the implementation of Aboriginal health policy	J Health Serv Res Policy	Empirical: qualitative descriptive; semi-structured interviews with 35 frontline health professionals	All sectors of the health system in Darwin, Alice Springs and remote Aboriginal communities, Northern Territory	Political/Organisational • Diverging views between AHWs and other professionals on scope of health care role including responsibility towards addressing social determinants of health
Peiris, D. et al., 2012 [37]	Building better systems of care for Aboriginal and Torres Strait Islander people: findings from the Kanyini health systems assessment	BMC Health Services Research	Empirical: theory-driven (‘Candidacy’) health system assessment involving group interviews with 37 health staff	7 health services (6 community-controlled and 1 government): two urban, one inner regional, two outer regional, and two remote, Queensland.	Social • Extension of AHW work beyond official hours Provider/Organisational • AHWs embody a community governance model and cultural brokerage role important but family obligations and kinship relations can sometime affect capacity to deliver health care
Dawson, A.P. et al., 2012a [13]	Aboriginal health workers experience multilevel barriers to quitting smoking: a qualitative study	International Journal for Equity in Health	Empirical: social-ecological lens using qualitative descriptive methods; in-depth interviews and focus groups	Urban, rural and remote health services in South Australia	Social • Widespread social acceptance and normalisation of tobacco use in the community Provider • Sense of professional responsibility for promoting smoking-cessation Organisational • Tacit acceptability of smoking in the workplace despite guidelines to the contrary Provider • Smoking as way to cope with stress of being ‘everything to everyone’
Dawson, A.P. et al., 2012b [54]	‘I know it’s bad for me and yet I do it’: Exploring the factors that perpetuate smoking in Aboriginal health workers - a qualitative study	BMC Health Services Research	Empirical: qualitative descriptive; in-depth interviews and focus groups	Urban, rural and remote health services in South Australia	Provider • Conflict between professional responsibility to promote smoking cessation and social norms around tobacco use. • High value placed on community relationships and trust Organisational/Provider • Smoking as a coping mechanism for stresses caused by job and financial insecurity including salary disparities and short term contracts, high staff turnover, lack of value and recognition by local and broader health system
Browne, J. et al., 2013 [55]	A qualitative evaluation of a mentoring program for Aboriginal health workers and allied health professionals	Australian and New Zealand Journal of Public Health	Empirical: qualitative evaluative; interviews (phone / face to face)	ACCHOs and state health services in Victoria	Political & Organisational • Uneven power dynamics with other health care providers
King, M. et al., 2013 [44]	Issues that impact on Aboriginal health workers’ and registered nurses’ provision of diabetes health care in rural and remote health settings	Australian Journal of Rural Health	Empirical: qualitative descriptive; ‘discussion schedule’ with 17 participants from nine health services (5 of whom were AHWs)	Two Aboriginal community controlled and seven mainstream health services in Far Western New South Wales	Political/Organisational • Non-recognition of qualifications and lack of incentives to develop and use new skills • Tension between Western models of health promotion work and culturally appropriate engagement with community Organisational • Perception of being ‘glorified taxi drivers’ transporting patients, with limited time available to apply expertise • Poor communication with health service managers perpetuates role confusion Provider • Cultural advocacy and brokerage work source of pride and sense of uniqueness
Rose, M. 2014 [36]	‘Knowledge is power’: Aboriginal Healthworkers’ perspectives on their practice, education and communities	Doctor of Education Thesis - University of Technology, Sydney	Empirical: Social ecological lens; qualitative descriptive; in-depth, semi-structured interviews with 9 health workers in diverse roles	A variety of communities (rural, regional, urban), New South Wales	Social • Being Aboriginal and part of the community gives strength in role Provider • Community advocacy and brokerage work key component of role Political/Organisational • Clear power differentials with non-Aboriginal colleagues, where AHWs are ‘bottom of the rung’ • Perception that further education could enhance power and status Provider • Co-workers’ and community perceptions of role sometimes divergent and a source of tension
Deshmukh, T. et al., 2014 [52]	‘It’s got to be another approach’: an Aboriginal health worker perspective on cardiovascular risk screening and education	Australian Family Physician	Empirical: qualitative descriptive; in-depth interviews	AMS in Western Sydney, New South Wales	Social • AHWs’ strong sense of connectedness and embeddedness in community Political/Organisational • Perceptions of being undervalued by health system and other health professionals
Jennings, W. et al., 2014 [34]	Yarning about health checks: barriers and enablers in an urban Aboriginal medical service	Aust J Prim Health	Empirical: qualitative descriptive; semi-structured interviews with clinical staff - 8 AHWs and 3 Aboriginal nurses	Urban community controlled AMS, Brisbane, Queensland	Organisational • Doctors perceived to have more authority by community members and also by other staff within health service Provider/Social • Cultural brokerage component of AHW role engenders community trust • Co-ownership approach to health between AHWs and community, and advocacy activity, key to role Provider • Cultural and social norms relating to gender, age & family background are in tension with some of AHWs’ professional obligations
Hengel, B. et al., 2015 [48]	Barriers and facilitators of sexually transmissible infection testing in remote Australian Aboriginal communities: results from the Sexually Transmitted Infections in Remote Communities, Improved and Enhanced Primary Health Care (STRIVE) Study	Sexual Health	Empirical: qualitative descriptive; in-depth interviews	Pirmary health centres in Queensland and Northern Territory	Social • Community connectedness promotes trust and improves access to clients. Provider/Social • Gendered cultural norms and cultural relationships influence appropriateness and ability to deliver care depending on gender and family connections of AHW staff
Lowell, A. et al., 2015 [47]	Supporting Aboriginal knowledge and practice in health care: lessons from a qualitative evaluation of the strong women, strong babies, strong culture program	BMC Pregnancy & Childbirth	Empirical: program evaluation; semi-structured interviews with 76 participants (incl 15 Strong Women Workers)’ analysis of reports	Five remote communities, Northern Territory	Provider/Political • Smoking ceremony for new babies and use of traditional medicine seen by AHWs as an important part of a broad and continuous process of promoting health and wellbeing that occurs throughout life - but this type of work and approach is under-recognised by broader health system and is sometimes at odds with policy. Political/Social • Under-recognition of cultural dimensions of health care increases community dependence on mainstream services under a biomedical model of service delivery, to the detriment of both AHWs and community members
Cosgrave, C. et al., 2016 [11]	Factors affecting job satisfaction of Aboriginal mental health workers working in community mental health in rural and remote New South Wales	Australian Health Review	Empirical: grounded theory study; semi-structured interviews	Rural and remote local health districts and community mental health services (NSW Health), New South Wales	Organisational • Role clarity difficulties impacting cross-professional collaboration • Perception among some non-Indigenous providers that AHWs are responsible for ‘anything Aboriginal’ • Perception among some non-Indigenous providers that Aboriginal clients always want to see Aboriginal health worker Political • Inequity in career pathways and remuneration as against qualifications and nature of work Social • Perception of being ‘everything for everybody’ • Tensions relating to service provision to clients with whom there may be family business or personal issues.
Kirkham, R. et al., 2017 [34]	Emotional labour and aboriginal maternal infant care workers: the invisible load	Women & Birth: Journal of the Australian College of Midwives	Empirical: phenomenological qualitative study; 30 in-depth interviews with staff and clients	Anangu Bibi Birthing Program, run at Port Augusta Hospital and involving Country Health, South Australia	Social • Connection with community enhances trust in AHW but simultaneously exposes them to emotional stress Provider • Experiences of tension between cultural and community obligations, and health service (clinical/institutional expectations. • Personal and professional roles blurred Organisational • Perceptions of other professionals’ lack of respect for, and misunderstanding of, AHW role and capacity Political • Institutional barriers to greater agency and professional aspirations of AHW
Conway, J. et al., 2017 [17]	The barriers and facilitators that Indigenous health workers experience in their workplace and communities in providing self-management support: a multiple case study	BMC Health Services Research	Empirical: multiple case studies; in-depth interviews	Rural and urban health centres including AMSs in five Australian states	Provider • Managing ‘dual relationships’ with health service managers and members of the community • Challenge of maintaining professional boundaries Social • Tensions regarding smoking - social pressure to smoke versus undermining role model capacity

None of the studies focused directly on AHW governance or accountability. However, nearly half of the included qualitative studies (*n* = 11) were focused on the experiences of AHWs in the workplace, including AHWs’ relationships with other health professionals and perspectives on their workplace. The other qualitative studies were evaluations of new programs or models of health care involving AHWs (*n* = 7), AHW perspectives on specific models of care (*n* = 2) and two studies focused on the challenges AHWs themselves faced in quitting smoking.

The organisational settings of the studies described in the included papers were varied and included those focused only on Aboriginal community controlled health services (*n* = 6), only government services (*n* = 3) or both community controlled and government services (n = 6). In 12 studies the health service operator was undisclosed. Study locations included urban, rural and remote communities across almost all Australian states and territories. South Australia had the largest representation of studies (*n* = 7), followed by the Northern Territory (*n* = 5), New South Wales (*n* = 4), Queensland (n = 3), Western Australia (*n* = 2) and Victoria (*n* = 1). A further four studies had an Australia-wide focus, with one focused jointly on Queensland and the Northern Territory.

### Findings of the review

We present the findings according to themes reflected in the data organised according to each of van Belle and Mayhew’s four dimensions of accountability – provider, organisational, political and social. We start with the social dimension, as this was described as being most important by AHWs themselves.

### Social dimensions of accountability

#### Serving community

AHWs’ sense of connection and belonging with their local communities was recognised in all papers as a key attribute of their role and was frequently discussed alongside a desire by AHWs to serve Aboriginal and Torres Strait Islander peoples within their localities. Connection with, and responsibility to, community was reflected on as essential to developing trust with patients and enabling AHWs to be effective in their work, as well as imbuing a profound source of satisfaction and pride. Specifically, building rapport with patients through knowledge of the community and recognising family connections was noted by AHWs to enable their health care role [34, 35], to give them strength and confidence [36], and to encourage community members to come to the service [36]. Similarly, community representation among health care staff in the form of AHW roles was seen to increase the ‘candidacy’ of Aboriginal and Torres Strait Islander communities to health services [37].

#### We are everything to everyone – Family ties and cultural obligation

AHWs’ connection with community, while described as essential to developing client and community trust and as a source of personal satisfaction and pride, also resulted in AHWs feeling like they are ‘everything to everyone’ [12, 38]. AHWs in multiple studies described feeling like they were never off-duty even after work hours [6, 37, 38]. A blurring of private and professional life was also described [35], with AHWs feeling a responsibility to help people in the community: ‘you’ve got to give them something, or help in some way’ [12]. The high community expectation experienced by some AHWs was also described [38, 39] and included fear of blame if and when something went wrong [35]. AHWs in one study described how such demands were personally challenging and could also create professional problems relating to clinical governance or confidentiality [35]. The close connection of many AHWs with their community and perceived greater susceptibility to community conflicts, grief, and blame were described as a significant source of emotional labour for AHWs [35]. Peiris et al. [37] and Genat [7] also noted that these responsibilities were often invisible to AHWs’ non-Indigenous professional colleagues. Nonetheless, in at least one study [34] there was a perception among AHWs that Aboriginal and Torres Strait Islander clients preferred to see doctors and saw them as having more authority to provide clinical advice than AHWs.

### Organizational dimensions

#### Power relations and workplace hierarchy

AHWs in a number of studies reflected on their position within a workplace hierarchy. In Jackson et al. [40], AHWs described how nurses in their health service positioned themselves as ‘in charge’ of AHWs, even when this was not their role. In Rose’s [36] account, an AHW working in a social work department of a NSW health service described her position in the hierarchy as ‘bottom of the rung’, with the non-Aboriginal social workers as her line managers. Genat [7] and Kirkham et al. [35] similarly describe the structurally-embedded nature of the clinical hierarchy, which requires AHWs to ‘go through the hierarchy to ask permission’ to deliver on their aspirations; this was seen to inhibit their ability to carry out important aspects of their work.

Workplace power differentials were seen to impact on AHWs’ autonomy, decision-making capacity and position within workplace teams [40], and to manifest in limited capacity of AHWs to use their experience and qualifications with Aboriginal clients [36]. However, a distinction was observed between AHWs’ experiences of workplace hierarchy in ‘mainstream’, or state or territory-run, health services versus in Aboriginal community controlled health services. For example, Aboriginal community controlled health services were noted as operating under the direction of an elected community board which was described as changing the relationship between AHWs and non-Indigenous colleagues [6]. Although not uniform, AHWs working within Aboriginal community controlled health services more frequently described their work as *with*, rather than *for*, the doctor – in contrast to a more hierarchical structure in government services [6, 41].

#### Role confusion and undervaluing of the role

A pervasive, system-wide, lack of understanding of AHWs’ role was discussed in various studies, and was reported as a barrier to AHWs working to their full capacity for the benefit of patients, broader society and their own professional satisfaction [6, 7, 12, 35, 38, 40, 42–46]. In many of these papers, the lack of understanding of the AHW role fed into (and was simultaneously compounded by) a general undervaluing of AHWs by non-Indigenous colleagues and was reflected in the different perspectives on the role as reported by AHWs and other professional health workers’ in the same service [40]. General undervaluing of AHWs was characterised in several studies by underutilisation of AHWs’ clinical skills and training. In three articles, for example, AHWs perceived that a significant proportion of their time was wasted being ‘glorified taxi drivers’ or ‘a taxi service’ for patients, limiting the time they had available to comprehensively apply their expertise [7, 35, 44]. AHWs in several studies further noted that non-Indigenous staff simply did not understand the cultural component of the role beyond the ‘interpretative’ function played by AHWs, and so found it difficult to characterise or place value on it [38, 47]. Genet [7] and Tregenza and Abbott [41] both noted that while some non-Indigenous health workers did value the cultural component of AHWs’ work, this was framed predominantly in terms of AHWs’ capacity to facilitate their own clinical practice, rather than as an important and intrinsic component of AHWs’ own profession.

Limited understanding of the AHW role was also seen to lead to a lack of recognition of the diversity of roles played by AHWs [6, 35], difficulties experienced by AHWs being accepted into workplace teams [12, 38], confusion about role boundaries [36, 48], exclusion of AHWs from clinical service delivery functions [42], and challenges in determining appropriate services for clients [43]. Mitchell et al. [38] reflected that the issue of skills utilisation was different for AHWs working within the ‘mainstream’ versus the community controlled sector: AHWs working within mainstream services are often tied to specific clinical areas or to non-clinical work such as transport; whereas AHWs working within Aboriginal community controlled health services experienced a broader clinical scope and have input into developing health programs. Rose [36] suggests that the widespread confusion around AHW roles arises in part from the complexity of policies that have informed development of the AHW profession (discussed further below). The pervasive lack of understanding of, and respect for, the AHW role among colleagues and institutions was described as leading to lower self-worth and stress among AHWs [35].

#### A holistic vs biomedical approach to health

Related to the issue of cultural value, several studies described the dissonance between their culturally-constituted and holistic philosophy of health and the dominant biomedical model of health operating in most service settings [35]. This dissonance operated at both an organisational and inter-personal level. At the organisational level, several papers reflected on the different philosophical approaches to health care between mainstream and Aboriginal community controlled health services [37, 38]. Mainstream services were seen to focus on a ‘disease-model approach’, and Aboriginal community controlled services were seen as being concerned with a broader ‘social model of health’ [38]. Mitchell et al. [38] reported that a ‘lack of cultural sensitivity’ was observed within mainstream services as compared with Aboriginal community controlled services. Peiris et al. [37] described positive experiences of an AHW working within an Aboriginal community controlled service, attributable in part to the governing board being composed of community members [37]. Mirroring the focus of the Aboriginal community controlled sector, the AHW role itself was described as representing a comprehensive primary health care approach underpinned by Aboriginal concepts of health [36].

At the individual and inter-personal level, tensions between the AHWs’, and nurses’ and doctors’, understandings of health were often discussed in relation to health promotion work. Culturally appropriate forms of community engagement were described by AHWs as being necessary for health promotion work to be effective in Aboriginal and Torres Strait communities, but these approaches were perceived by many AHWs to be under-recognised within established models of health promotion and were therefore not supported or understood by other health professional colleagues [44]. Similarly, effective engagement with community was seen by AHWs in two studies as requiring ‘going out’ into community to talk with people in situ, often outside of the clinic and outside of working hours [44, 49].

#### Cultural brokerage

Despite reported undervaluing of cultural aspects of the AHW role, cultural brokerage was recognised by multiple actors as a defining feature of AHWs’ role [6, 36, 37]. This part-interpretative, part-advocacy function required AHWs to draw on their understanding of community issues and priorities to enable colleagues and the broader system to respond to Aboriginal and Torres Strait Islander peoples’ needs. Tangible tasks related to cultural brokerage included cultural mentorship of non-Aboriginal and Torres Strait Islander colleagues [6], reflecting Aboriginal patients’ concerns as part of their own concerns [34], giving voice to patients who may feel reticent to access the service [50] and speaking up with confidence when government health services were taking an approach that’s not ‘our way’ [50]. Peiris et al. also noted an advocacy function played by an Aboriginal community controlled service on behalf of Aboriginal clients in liaising with the local hospital, operationalised through their AHW staff [37]. In their wide-ranging survey of AHWs in the Northern Territory, however, Tregenza and Abbott [41] noted that doctors and nurses placed more emphasis on the cultural brokerage role than AHWs did, as it became a critical enabler of their own (clinical) practice, rather than as a platform for strengthened recognition of the centrality of Aboriginal and Torres Strait Islander peoples’ conceptions of health.

#### Responsible for ‘anything aboriginal’

Several studies in both urban hospital and remote primary care settings reflected on the way non-Indigenous providers drew a connection between AHWs’ Aboriginality and a particular responsibility or expectation to assist all Aboriginal patients irrespective of presenting problem [12, 39, 41, 46]. AHWs reported a sense of duty when a patient was identified as Aboriginal, which included a responsibility to look after the patient’s families and relatives [46]. However, the assumption by non-Indigenous staff, or health organisations, that AHWs were responsible for ‘anything Aboriginal’ within their service, was also described as intrusive and as representing an inadequate grasp of the AHW role and client needs [12, 46]. This assumption included a perception held by non-Indigenous staff that ‘Aboriginal clients…always want to see an Aboriginal worker’ (Cosgrave et al. 2016), a concern echoed by Williams et al. [46] as they described a situation in which all Indigenous clients in a hospital department were referred to the Aboriginal health team, regardless of the prevailing health concern or clients’ preferences. Taylor et al. [47] similarly reported the experience of an AHW who, as the only Aboriginal staff member within a hospital department, was continually approached for all socio-cultural needs of Aboriginal clients, increasing the ‘social worker’ components of their role while limiting their ability to use their clinical skills outlined in their job description. Harris et al. [42] and Tregenza and Abbott [41] both highlight the problematic assumption that AHWs are automatically and sufficiently culturally skilled or experienced to manage complex demands relating to their cultural mentorship or brokering role. Several papers additionally described the demanding responsibilities experienced by AHWs and high expectations from the system in remote area services, relating to AHWs’ navigation of cultural and linguistic challenges where English was a second or third language [39, 41].

### Political dimension

#### Career structure

In a number of studies representing views across several decades, AHWs reported feeling as if they were ‘jack of all trades but master of none’, with insufficient professional standing or opportunities for career progression or mobility. Tregenza and Abbott [41] reported widespread frustration among AHWs working in both government and Aboriginal community controlled sectors in central Australia, with the lack of clarity around roles, responsibilities and opportunities to progress professionally. Reporting findings from a mixed methods study that included a questionnaire, Dollard et al. [51], reported that two-thirds of the 74 AHWs surveyed reported feeling dissatisfied with their status compared to other staff in their organisation. Reasons given in open-ended responses included lack of recognition as a professional, and inequality in relation to pay and work conditions when compared to other health professionals. Mitchell et al. [38] reflected on the lack of agreed national, or even state-based scopes of practice for AHWs as an ongoing barrier to the development of the AHW role. This barrier may still be relevant to AHWs despite the 2012 introduction of the National Registration and Accreditation Scheme for Aboriginal and Torres Strait Islander Health Practitioners, due to its narrow focus on registered practitioners.

Reflecting on the range of policies that have influenced the development of the AHW role (see Table 1) over several decades, Rose [36] noted that the complexity of multiple recommendations emerging from Commonwealth and state governments as well as from the Aboriginal community controlled health sector, was compounded by the jurisdictional and organisational differences between states and territories, and between individual community controlled health services, in policy implementation, with stasis a frequent outcome.

#### Professional development: ‘Knowledge is power’

Education was described in a number of papers as offering the possibility of increasing the power and agency of AHWs in the workplace [36, 39, 40]. Several papers from the 1990s, [40, 41, 52] however, described comparatively limited opportunities for further education available to AHWs. Hecker et al. [52] reflected that access to training and educational opportunities for AHWs at that time were largely dependent on the motivations of the individual. The same paper also reported low standards of training and lack of skills in English literacy and numeracy that created barriers to accessing those educational opportunities that did exist. More recent papers included accounts that suggest some improvements in access to education. Rose [36] reported the account of one AHW who received training as an Aboriginal Health Education Officer, describing how her education gave her additional confidence and knowledge to meet both community and co-workers’ expectations, reflecting that ‘knowledge is power’. However, without structural reforms relating to AHW career structure, King et al. [44] described a perception among some AHWs that more recently-acquired qualifications were perhaps not worth the effort, given that after gaining these they were offered no specific role or responsibility to use their new skills.

### Provider dimension

#### Straddling different cultures

Multiple papers highlighted an overarching distinction, and often tension, between the cultural and community obligations and clinical and administrative aspects of the AHW role [34, 35, 40]. AHWs in one study reflected that the AHW approach, in contrast to other health professional roles, was one of ‘co-ownership’ of health by both themselves and their community, because patients are ‘our people’ [34]. In Deshmukh et al. [53] an AHW similarly reflected that ‘as AHWs we are still the community’. Lloyd et al. [45] contrasted the perspectives of AHWs with those of nurses on the scope of their health care role, with nurses emphasising boundaries between ‘the clinic’ and individual responsibility, and AHWs recognising the social and economic determinants of the diseases presenting in the clinic. The clinical aspects of the role were described in one study as ‘largely institutionalised’, and as favouring ‘objectivity and maintenance of professional boundaries’ [35]. Reflecting a policy manifestation of the tensions, a situation was described in a Northern Territory government program evaluation in which Aboriginal ‘Strong Women Workers’ were helping Aboriginal women to give birth in local communities, against Departmental policy and without remuneration, because they believed this was better for the mother and baby [49].

The need to straddle the two cultures was also described at a more interpersonal level with the need for AHWs to constantly manage the expectations of non-Indigenous health professionals including managers [18]. Reflecting on tensions between the community-oriented versus clinic-oriented framing of their role, AHWs in Rose [36] emphasised their community role as their ‘real’ role, involving informal discussion with community members about complex issues, as compared with more ‘formal referrals’ or clinic-based tasks. An AHW in a much earlier study similarly expressed their prioritisation of community responsibilities if any tensions were apparent [40]. In some studies, however, AHWs reported frustration with the presumption that they should only or predominantly focus on culturally-informed counselling duties, expressing a desire to develop and/or better utilise their clinical skills [7, 12, 18].

Beyond these overarching issues, many articles reflected on specific tensions between cultural and professional obligations. The challenges of straddling the two cultures was described in two papers exploring the reasons for high rates of smoking among AHWs responsible for chronic disease services, which found that smoking practices were reinforced by AHWs’ embeddedness in communities *and* health services where smoking was normalised or at least practiced socially. This was, as well, found to be a response to the stress arising from the multiple demands of their role [13, 54]. McMasters et al. [39] also described a clash between some non-Indigenous or even institutional-based expectations that AHWs should be giving lifestyle advice to Aboriginal clients, and AHWs’ own culturally-informed perspective that giving such advice was likely to be interpreted by Aboriginal clients as a criticism and interfering.

In Jackson et al. [40], AHWs described their experiences balancing complex lines of responsibility, and instances of incompatibility in their professional role with cultural expectations relating to gender, age and kinship. Jennings et al. [34] also reported female AHWs feeling reluctant to discuss some lifestyle modification behaviours with male, and particularly elder male, clients, or with males from differing cultural groups. Hengel et al. [48] described barriers to patient access relating to the nature of cultural relationships involving AHWs and members of the community. In one study, being based in a rural health service was seen to exacerbate challenges faced by AHWs in navigating between Aboriginal and health service culture, since staff shortages in the area meant that AHWs were expected to see patients with whom there were tensions due to family business, cultural taboo or personal dynamics [12]. These cultural challenges were also seen to arise within the workplace, such as where older AHWs carried their role as elders into their relations with other staff, with the potential for conflict [38].

#### Partnership models and improved communication

Several papers described the benefit to workplace dynamics and ultimately healthcare delivery of the implementation of partnership models between AHWs and their non-Aboriginal colleagues [6, 55]. One of these described a peer-mentoring model between Aboriginal and non-Aboriginal health professionals, which was found to improve communication between staff and facilitate more collegial relationships [55]. A need for better communication between health service managers and AHWs was identified in another study that was seeking to address poor communication by managers about the objectives and targets of projects, and to break down organisational hierarchies [44]. One AHW described the increased patient trust developed towards midwives following initiation of a two-way partnership model between AHWs and midwives, within which the roles were treated as ‘mutually equivalent’ [50]. However, another program focused on implementing two-way learning based on cultural knowledge of AHWs highlighted a number of challenges in both design and implementation of the model, including lack of consensus on how two-way learning could lead to improved outcomes and on what mechanisms are needed to support the integration of AHWs into a particular practice environment [42].

## Discussion

We conducted an adapted systematic review of the published literature to map the evidence relating to AHWs’ accountability relations in the Australian health system. We found few studies with a primary focus on AHW governance and none with a primary focus on AHWs’ accountability relationships. Nonetheless, data from a range of empirical studies and AHW-authored commentaries did provide evidence of AHWs’ workplace experiences and some insight into the accountability eco-system in which this unique cadre operate.

AHWs experience multiple and overlapping accountabilities in all four of the dimensions proposed by van Belle and Mayhew [26]. Reflecting accountabilities in the social dimension, AHWs’ sense of connection and belonging within their community was frequently discussed. In several articles [7, 37, 41] AHWs reported feeling a strong sense responsibility for ensuring that the health service was accessible to and safe for ‘their people’, and described their role as a critical mediator in ensuring such access. While described as essential to developing trust and as a source of pride, connection with community and the obligations it implied also came at a cost. AHWs in a number of studies reported being constantly on duty and having a sense of being ‘everything to everyone’. These themes are reflective of findings from several recent studies of CHWs in both low and middle-income settings which have highlighted the paradox between the value of CHW connectedness to community, and the difficulties of simultaneously managing community expectations and demands [18, 56–58].

Obligations from the social domain overlapped with those in both the organisational and provider dimensions of accountability. AHWs in multiple studies reported having to straddle concurrent cultural obligations including those relating to gender, age and kinship, and the expectations of non-Indigenous colleagues and managers rooted in Western models of clinical governance and management. Biomedical dominance combined with lack of role clarity were linked to pervasive system-wide misunderstanding of AHWs’ role and responsibilities – particularly the cultural components – which in turn acted as a barrier to AHWs working to their full capacity for the benefit of patients, broader society and their own professional satisfaction. Tensions between these ‘horizontal’ organisational accountabilities and the ‘downwards’ accountability to communities were highlighted in descriptions of dissonance between AHWs’ cultural and professional identities as they tried to negotiate a path between community expectations, requirements of their often bio-medically oriented workplace, and their own professional aspirations. These tensions are arguably exacerbated by prevalent policy expectations that AHWs can address or improve health outcomes caused by profound, often inter-generational structural inequities linked to poverty and social marginalisation.

Despite reports of AHWs being ‘everything to everyone’ and a ‘jack of all trades’, the literature also demonstrated that AHWs often struggled to gain sufficient educational attainment or professional opportunities in order to be able to demonstrate mastery of their practice that would enable them to progress in their career. Indeed, the reviewed literature highlighted the ambiguity of the AHW role and the large gap between the idealised capabilities and presumed centrality of the role within health provider teams, and the experienced reality of multiple and sometimes mutually-exclusive obligations to community and the system. Power imbalances in AHWs’ workplaces stemming from clinical hierarchies and vertical health administration structures meant that there were limited opportunities to discuss or mediate the above-mentioned tensions. AHWs’ felt their professional influence and status were limited, many reporting how the same power dynamics and structures, rooted in the biomedical approach of Australia’s mainstream health system, in fact challenged and undermined their own conceptualisation of health and wellbeing. Perceptions of being under-valued and poorly supported by the health system at the macro and meso (state based career structures) and micro (health facility) levels, were a common theme and mirror experiences elsewhere [59, 60].

The review demonstrates evidence of complexity in AHWs’ accountability relationships in literature spanning several decades. Yet this study also highlights the large gaps in theoretically-informed and systematic investigations of accountability relations and related issues including the power dynamics that underpin both governance and accountability arrangements for AHWs in the Australian health system. While various studies made reference to the ‘dual role’ and AHWs’ straddling of ‘two cultures’, most were descriptive in nature and defaulted to recommending improved role clarity and clinical (often specialist) training for AHWs as the remedy. Few articles reflected meaningfully on the inherent tensions of a role that is, at its core, culturally-constituted, but located within a service environment shaped by vertical power relations and (in many settings) a Western biomedical model of healthcare. Nor, despite some observations regarding the challenges experienced by female AHWs, did we find evidence of gender analysis in relation to this highly feminised cadre of providers. Writing about CHW programs more generally, others have noted that questions remain relating to the influence of shared culture and gender on CHW program effectiveness, and the most appropriate methods of integrating culture into program design and training [61].

Tregenza and Abbott [41] and Genat et al. [7] both explored these issues in greater depth, albeit in very different settings and drawing on data collected in the mid- and late 1990s respectively. Yet several significant policy shifts have occurred in the AHW profession (see Table 1) and in the policy landscape of the Australian health system (e.g. introduction of the Close the Gap initiative [5]) since this time. Up-to-date investigation of the governance arrangements and accountability relationships under the contemporary policy architecture is required, including theoretically-informed work, such as the study described by Peiris et al. [37], which seeks to understand whether and how governance arrangements in both mainstream and community-controlled health services can better accommodate and support this complex role.

## Conclusion

Health inequity among Aboriginal and Torres Strait Islander peoples and non-Indigenous Australians is pronounced and for several decades AHWs have been identified within commonwealth and state policies as important mediators of access to culturally safe care and as a strategy to help close the gap between Indigenous and non-Indigenous health outcomes. Understanding how the governance and accountability relationships of AHWs influence their ability to address health inequities experienced by Aboriginal and Torres Strait Islander peoples has both ethical and policy significance. This review provides some evidence for how AHWs operate within a complex accountability eco-system, flagging multiple and overlapping accountabilities that influence job satisfaction, performance and willingness to remain in service. However, systematic and up-to-date evidence relating to the nature and impact of AHWs accountability relationships in the varied political (state), geographic (urban/rural) and service (government / community controlled) domains remains limited. More theoretically-informed research to examine the nature of AHW accountabilities and how they are formed within both mainstream and Aboriginal community-controlled governance models is urgently needed to inform workforce policy and ultimately improve health service provision to Aboriginal and Torres Strait Islander peoples.

## Abbreviations

AHP: Aboriginal (and Torres Strait Islander) Practitioner
AHW: Aboriginal (and Torres Strait Islander) Health Workers
CHW: Community Health Worker
NRAS: National Registration and Accreditation Scheme
QLD: Queensland
UHC: Universal health coverage
